# A GPS-Referenced Wavelength Standard for High-Precision Displacement Interferometry at λ = 633 nm

**DOI:** 10.3390/s23031734

**Published:** 2023-02-03

**Authors:** Ulrike Blumröder, Paul Köchert, Thomas Fröhlich, Thomas Kissinger, Ingo Ortlepp, Jens Flügge, Harald Bosse, Eberhard Manske

**Affiliations:** 1 Institute of Process Measurement and Sensor Technology, Technische Universität Ilmenau, 98693 Ilmenau, Germany; 2 Precision Engineering Division, Physikalisch Technische Bundesanstalt (PTB), 38116 Braunschweig, Germany

**Keywords:** GPS-disciplined oscillator, optical frequency comb, traceability, ultrastable laser, displacement interferometry, nanopositioning and nanomeasuring machine (NPMM)

## Abstract

Since the turn of the millennium, the development and commercial availability of optical frequency combs has led to a steadily increase of worldwide installed frequency combs and a growing interest in using them for industrial-related metrology applications. Especially, GPS-referenced frequency combs often serve as a “self-calibrating” length standard for laser wavelength calibration in many national metrology institutes with uncertainties better than *u* = 1 × 10^−11^. In this contribution, the application of a He-Ne laser source permanently disciplined to a GPS-referenced frequency comb for the interferometric measurements in a nanopositioning machine with a measuring volume of 200 mm × 200 mm × 25 mm (NPMM-200) is discussed. For this purpose, the frequency stability of the GPS-referenced comb is characterized by heterodyning with a diode laser referenced to an ultrastable cavity. Based on this comparison, an uncertainty of *u* = 9.2 × 10^−12^ (*τ* = 8 s, k = 2) for the GPS-referenced comb has been obtained. By stabilizing a tunable He-Ne source to a single comb line, the long-term frequency stability of the comb is transferred onto our gas lasers increasing their long-term stability by three orders of magnitude. Second, short-term fluctuations-related length measurement errors were reduced to a value that falls below the nominal resolving capabilities of our interferometers (Δ*L*/*L* = 2.9 × 10^−11^). Both measures make the influence of frequency distortions on the interferometric length measurement within the NPMM-200 negligible. Furthermore, this approach establishes a permanent link of interferometric length measurements to an atomic clock.

## 1. Introduction

The demand for precise frequency measurements in the optical domain, the coalescence of decades of research and development in the field of pulsed laser systems, laser spectroscopy and nonlinear material interaction, as well as new findings on pulse propagation in micro structured optical fibers have led to the rise of the optical frequency comb technology at the turn of the millennium [1,2,3,4,5,6,7]. An optical frequency $\upsilon_{n}$ within the spectrum of a mode-locked pulse laser is defined by its respective mode number *n*, the repetition rate *f*_Rep_, and carrier envelope offset (CEO) frequency *f*_CEO_ [8]:


(1) $$\upsilon_{n}=n\cdot f_{\mathrm{Rep}}\;\pm \;f_{\mathrm{CEO}},$$

Both comb parameters *f*_Rep_ and *f*_CEO_ are usually in the rf domain, thus phase locking them to an atomic clock provides a direct link of an optical frequency to a primary frequency standard and consequently, the SI meter definition. In this way, ultraprecise, traceable optical frequency measurements can now be performed with unprecedented small experimental effort and cost. Within the revision of the practical realization of the definition of the meter by the Comité international des poids et mesures (CIPM) in 2002, it was considered

“… that new femtosecond comb techniques have clear significance for relating the frequency of high-stability optical frequency standards realizing the SI second, that these techniques represent a convenient measurement technique for providing traceability to the International System of Units (SI) and that comb technology also can provide frequency sources as well as a measurement technique.”

[9] (p. 182), [10] (p. 104).

Furthermore, the CIPM recognized “comb techniques as timely and appropriate” [9] (p. 182), [10] (p. 104) and urged ”national metrology institutes and other laboratories to pursue the comb technique to the highest level of accuracy achievable and also to seek simplicity so as to encourage widespread application” [9] (p. 182), [10] (p. 104).

Therefore, frequency combs quickly started to complement and replace common frequency standards, such as the iodine-stabilized He-Ne laser in national metrology institutes [11,12] and spurred research in a wide range of high-precision metrology applications [2,4]. Especially GPS-referenced frequency combs provide a cost-effective alternative to an atomic clock and can serve as a “self-calibrating” wavelength standard for laser wavelength calibration where an uncertainty of 10^−12^ appears to be sufficient [13,14]. Their unique features also turned them into a versatile tool for precise interferometric distance measurements that are playing a crucial role for the advance of precision engineering and nanotechnology [15]. Especially for applications in nanotechnology, length metrology has to face tremendous challenges due to increasing demands on measurement range, accuracy, traverse speed, and traceability (see e.g., [16]). Herein, the combination of state-of-the-art interferometric methods with optical frequency combs has put the term “high-precision” to the next level and created new possibilities for absolute and incremental interferometric length measurements.

Absolute distance measurements based on multiwavelength and dispersive interferometry benefit from the broad bandwidth of frequency combs and the high temporal coherence of individual comb lines as well as their traceable, absolutely known frequencies when a comb is referenced to an atomic clock [17,18,19,20,21,22,23,24,25]. For applications in absolute distance measurements, one option is to use the comb directly in a Michelson interferometer configuration with a spectrally resolved detection scheme [17,18,19]. Therein, the application of a high-resolution spectrometer has made a spectral resolution to the level of individual comb modes for a comb repetition rate of 1 GHz possible and a combination of spectral interferometry and multiwavelength, homodyne interferometry was applied to determine distances within an unambiguity range of 15 cm and a standard deviation of 28 nm compared to conventional homodyne displacement interferometry [19]. Lately, this method has been evaluated with an incremental He-Ne laser interferometer demonstrating an agreement in the micrometer range over distances up to 50 m [20].

A second option is to utilize individual modes of the comb spectrum as a traceable, stable reference for multi-wavelength interferometry allowing to create multi-wavelength sources with high output power [21,22,23]. With this approach recently a comb-referenced four-wavelength-source for distance measurements up to 3.8 m with a combined relative measurement uncertainty of 1.62 × 10^−8^, that was mainly determined by the uncertainty of the empirical compensation of the refractive index was demonstrated and suggested for traceable, precision distance measurements for mass-production of optoelectronic devices [23].

In this contribution, we introduce the concept of a comb-referenced metrology laser at 633 nm for high-precision homodyne displacement interferometry in a nanopositioning and nanomeasuring machine (NPMM) with a maximum positioning range of 200 mm in a single measurement axis. NPMMs combine high-precision length measurement and positioning of macroscopic objects at nanometer level [26]. Their development was driven by the need for traceable, dimensional metrology with nm-accuracy for nanotechnology and semiconductor industry at the turn of the millennium [26]. To this day, different solutions for high-precision full 3D measurement of micro and nano parts based on coordinate metrology have been developed [27]. At the TU Ilmenau, the NMM-1 with a measurement volume of 25 mm × 25 mm × 5 mm was developed at the turn of the millennium [28]. Today, this system is commercially available [29] and it is applied in national metrology and research institutes for the measurement of micro- and nanostructures or international comparisons of step height standards [30,31]. The demand for extended measurement volumina has recently led to the development of the NPMM-200 providing a measurement range of 200 mm × 200 mm × 25 mm with a one-point positioning stability and repeatability of less than 2 nm for lateral measurements over the whole measurement area and a standard deviation of 20 pm for vertical repeatability measurements of a 5 mm step height standard, resulting in a relative resolution of 10 decades considering the full measurement range of 200 mm [26,32]. Currently, one machine is installed at the TU Ilmenau and another one at the Institut für Technische Optik (ITO) in Stuttgart, where it is applied e.g., for the characterization of freeform optics [33]. The positioning performance of the NPMM-200 sets new demands on the frequency stability of the laser sources used for the underlying interferometric length measurement systems. Currently, polarization stabilized He-Ne gas lasers that still belong to the most widely spread laser sources for precision distance measuring interferometry are deployed [34]. Their typical relative frequency deviation of 2–6 × 10^−9^ over a 24 h measurement results in a measurement error of 0.4–1.2 nm if a maximum measurement range of 200 mm is considered [35]. Additionally, degradation due to the aging process of the gain medium cause verifiable drifting in the center frequency during lifetime operation of He-Ne lasers [36,37]. Niebauer et al. believe that an effective accuracy of 1 × 10^−9^ is technically feasible with two calibrations per year [36]. Thus, they have to be periodically measured against a Bureau International des Poids et Measures (BIPM)-compliant frequency standard to guarantee full traceability of interferometric length measurements within the NPMM-200 to the SI meter definition [35].

In this work, we will discuss how a He-Ne laser source permanently tied to a GPS-referenced frequency comb acts as a traceable wavelength standard for displacement interferometry in the NPMM-200 improving the frequency stability of the He-Ne metrology lasers by three orders of magnitude. The approach builds up a permanent link between the interferometric length measurement and an atomic clock and thus the SI second.

The paper is organized as follows. The experimental arrangement of the comb-referenced metrology laser will be presented in Section 2. In Section 2.1, we describe the initial length measurement system of the NPMM-200 and the traceability chain when using polarization-stabilized He-Ne lasers. Based on this configuration we will derive the necessary properties of a traceable laser source to be integrated into the positioning system of the NPMM. In Section 2.2, we introduce the metrological fundament for creating ultrastable traceable frequencies at 633 nm. The backbone is a GPS-referenced frequency comb that is accompanied by an Optical Reference System (ORS), consisting of a diode laser locked to a high-finesse cavity. Afterwards we introduce the practical implementation of a comb-referenced He-Ne laser in Section 2.3 and finally describe the methodology for characterizing the frequency stability of the respective laser sources in time domain in Section 2.4. The main results of our work will be presented and discussed in Section 3. Therein, Section 3.1 contains a frequency stability analysis of the GPS-referenced frequency comb and in Section 3.2, we present the results of the comb-stabilized He-Ne source. We discuss the consequences of this stabilization regime on the traceability chain and the impact on length measurement errors within the NPMM-200. In Section 4, we finally summarize and conclude our work and give an outlook on upcoming experiments and developments.

## 2. Experimental Configuration and Methods

### 2.1. State of the Art Length Measuring System of the NPMM-200

**Figure 1:**
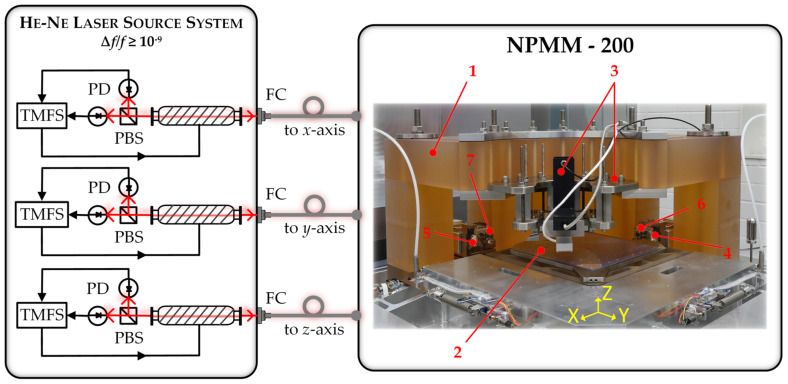
Setup of the NPMM-200 and its He-Ne laser source for three interferometer axes. The abbreviations in the left picture denote: TMFS—two-mode frequency stabilization, PD—photodiode, PBS—polarizing beam splitter, FC—fiber coupling. The numbers in the right picture correspond to: 1—metrology frame, 2—Positioning stage with corner mirror plate, 3—Sensor mount with nano probe system, 4, 5—Interferometer *x*, *y*-axis, 6, 7—Autocollimators (angle sensors). The interferometer of the z-axis is not visible. The base plate below the metrology frame additionally carries the guiding and drive system of the *x*-, *y*-, and *z*-axis (see details in [32,40]).

The basic metrological concept of the NPMM-200 is presented in Figure 1 and was lately described in [32]. Further details on the underlying design principles, the drive and guidance system as well as the respective control loops can be found in [38,39,40,41] and the references given therein. The NPMM-200 applies a high-precision interferometric measuring system to determine the lateral and vertical position of the moving stage and angular deviations of the guidance system [32].

**Figure 2:**
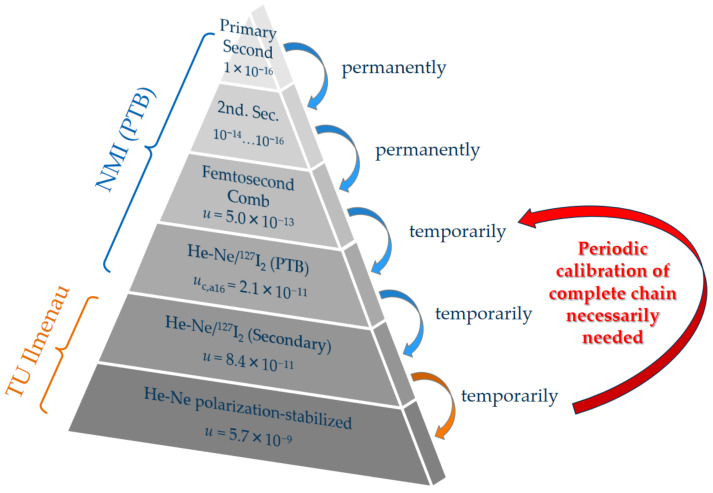
Scheme of a possible traceability chain for laser wavelength calibration deploying iodine-stabilized He-Ne lasers. The given uncertainties were taken from the literature as given within the text.

The measuring scale of the displacement interferometers of the *x*-, *y*-, and *z*-axis is determined by the wavelength *λ* of the corresponding laser which is a function of the frequency *ν* of the laser and the refractive index of the ambient medium *n*_ambient._ Therefore, changes of the laser wavelength are directly considered as length measurement errors Δ*L*_Meas_. The uncertainty in the determination of the laser frequency *ν* and *n*_ambient_ thus finally limit the precision of length measurements in the NPMM-200. For measurements under ambient conditions, the environmental parameters (pressure, temperature, and humidity) are permanently monitored and the refractive index is calculated from an updated version of the Edlén formula [42]. Nevertheless, the compensation of the refractive index due to these empirical equations is usually limited to an uncertainty of 10^−7^–10^−8^, mainly caused by uncertainty contributors resulting from the determination of ambient conditions and the air composition [43,44]. Therefore, the NPMM-200 can be additionally operated under vacuum down to 1 mbar to reduce the influence of the refractive index. The signal processing unit of the interferometers utilizes 16-bit A/D-converters for a demodulation of the interferometer signals [40]. Their resolving capability was determined according to [45] (see Table 1 in [45]) with a value of 5.72 pm, assuming an analog-to digital quantization of 16 bit and a rounding error of 16 bit for the arctan function. The interferometers are fiber-coupled and deploy frequency-stabilized He-Ne lasers to minimize the influence of frequency fluctuations on the length measurement. Each of the lasers is independently stabilized by a two-mode comparison technique [46]. Frequency changes can be achieved by varying the resonator length due to increasing the current through the silver heating coil around the He-Ne tubes (see Figure 1). Currently, each of the interferometer axes is powered by one He-Ne laser. These lasers are embedded in a compact, enclosed laser module containing the laser control and stabilization electronics. The He-Ne lasers provide a maximum output power of 700–900 µW per interferometer axis after fiber coupling. The laser module is placed outside the NPMM-200 together with the rest of the NPMM-200 control electronics to avoid heat dissipation from the laser sources (maximum electrical power consumption 60 W) into the NPMM-200 [40]. Considering a typical measurement interval of 24 h, the polarization-stabilized He-Ne lasers used within the NPMM-200 showed maximum frequency deviations of 1.2–2.7 MHz (relative frequency deviation Δ*f*/*f* = 2.5 × 10^−9^–5.7 × 10^−9^) from the mean value resulting in a measurement error of 0.5 nm–1.1 nm for a maximum measurement range of 200 mm [35]. Those results are in accordance with other long-term measurements of internally stabilized He-Ne lasers [47]. Additionally, changes of the center frequency and the shape of the gain profile directly influence the absolute frequency value of the polarization stabilized He-Ne lasers since the control signals for frequency stabilization are directly derived from the gain profile. Therefore, their lifetime wavelength stability is usually limited to 2 × 10^−8^ [35,47]. For a measurement range of 200 mm, the corresponding frequency changes of 5–10 MHz would result in a systematic length measurement error of 2–4 nm [35]. To overcome these errors during long-term operation of the NPMM and guarantee full traceability of the length measurement to the SI meter definition, the absolute frequency value of the metrology lasers has to be periodically measured against a BIPM-compliant frequency standard. For the purpose of frequency calibration, the iodine-stabilized He-Ne laser operating at a wavelength of 633 nm with an uncertainty of *u* = 10 kHz (2.1 × 10^−11^) has been mainly used in the visible spectrum for the practical realization of the SI meter definition [48,49]. However, this level of uncertainty can normally only be stated by national metrology institutes (NMI). In Figure 2, a possible configuration for laser vacuum wavelength calibration including iodine-stabilized He-Ne lasers is presented. To demonstrate the hierarchy between the primary realization of time and the realization of the meter definition by using frequency measurements all possible steps with their respective reported uncertainties are included. National metrology institutes (NMIs) realize the unit of time with the highest accuracy. For the best of these primary frequency standards, relative standard uncertainties in the order of 10^−16^ have been reported [50,51]. The NMIs often hold secondary representations of the second such as optical strontium clocks or rubidium oscillators whose frequencies have to be measured against the primary standards leading to relative uncertainties between 10^−14^ and 10^−16^ [50]. Other commercially available frequency standards as cesium (Cs) clocks or hydrogen masers are often redundantly operated from the NMIs for time-service applications [50]. They can furthermore be applied as rf-references for optical frequency combs, determining their uncertainty [52] in case of, e.g., cesium clocks to a level of 5 × 10^−13^ [50].

Although optical frequency combs are increasingly applied for laser vacuum wavelength calibration, the iodine-stabilized He-Ne lasers are still used for frequency calibration of commercial polarization-stabilized He-Ne lasers and for additional performance tests of GPS-referenced frequency combs (see e.g., [53]). Nevertheless, their uncertainty of 2.1 × 10^−11^ can increase to 8.4 × 10^−11^ for systems operating as a secondary standard in industry or research labs [54]. When measuring a polarization-stabilized against an iodine-stabilized He-Ne laser the uncertainty of the frequency calibration is given by the frequency stability of the specific laser under test [47]. For polarization-stabilized lasers, relative standard uncertainties (Typ B, k = 2) between 1.3 × 10^−9^ and 5.7 × 10^−9^ for a 24 h-measurement interval have been reported [47]. Based on this uncertainty hierarchy of frequency calibration and the described state-of-the-art configuration of the length measurement system, the following requirements for an ultrastable, traceable next generation metrology laser for displacement interferometry in the NPMM-200 can be deduced:

For an easy integration into the current length measurement system of the NPMM-200, the next generation metrology laser should be in the VIS near 632.8 nm and provide enough output power (>500 μW) to be suitable for fiber coupling. To practically eliminate the influence of frequency distortions on the length measurement in NPMMs with extended measurement areas of several hundred millimeters, it is desirable that the short-term related length measurement errors fall below the nominal resolution limit of the homodyne interferometers. For the NPMM-200, this limit can currently be set to 2.86 × 10^−11^ (5.72 pm at 200-mm measurement range). Taking into account a resolution enhancement in A/D conversion and a possible further extension of measurement ranges up to 1 m, there will prospectively be even a frequency stability of 10^−12^ desirable. Ideally, this frequency stability should be available irrespective of integration time. Finally, to control the absolute frequency of the laser source and thus completely trace back the interferometric length measurements, the metrology laser should ideally provide a permanent link to a primary frequency standard [55]. These requirements cannot be fulfilled with the currently used polarization-stabilized He-Ne lasers. In the following sections, we will thus introduce the new comb-based concept for interferometric distance measurements within the NPMM-200.

### 2.2. Metrological Basis for Generation of Ultrastable, Traceable Optical Frequencies at 633 nm

The commercial availability of traceable, ultrastable optical frequency sources, that have for a long time only been accessible to national metrology institutes, paved the way to create a new metrological base for ultraprecise frequency measurements at the Institute of Process Measurement and Sensor Technology (IPMS) at the TU Ilmenau. The key is a GPS-referenced frequency comb providing exceptional long-term frequency stability and direct traceability to the SI unit second. This fundamental wavelength standard is complemented by an optical reference system (ORS) that is based on a diode laser referenced to an ultrastable high-finesse cavity. Such a laser system provides an exceptional short-term stability and can be used as stand-alone system or as an optical reference for the frequency comb [56]. In this section, we describe the configuration of both laser systems.

#### 2.2.1. GPS-Referenced Frequency Comb (OFC)

**Figure 3:**
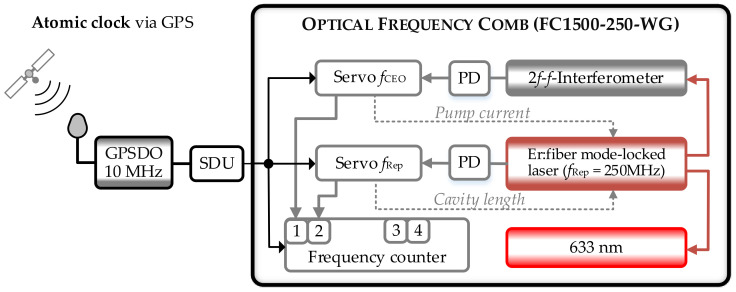
Structure of the GPS-referenced frequency comb at the IPMS. The abbreviations denote: OFC—optical frequency comb, GPSDO—GPS disciplined oscillator, SDU—signal distribution unit, PD—photodiode. The numbers 1–4 correspond to the respective frequency counter channels.

The GPS-referenced frequency comb at the IPMS is a commercial system from Menlo System (model: FS 1500-250-WG, Figure 3). The comb parameters are phase locked to the frequency output of a GPS-disciplined oscillator (GPSDO). The GPSDO uses the time signal from the GPS (or other GNSS) satellites to steer the frequency output of a local oscillator [13]. The local oscillator of the GPSDO provides the short-term stability (in this case τ ≤ 100 s) whereas the long-term stability and accuracy of the local oscillator is determined by the GPS signals that are directly traceable to the Universal Time Coordinated (*The United States Naval Observatory*) UTC (*USNO*) [13]. To transfer the accuracy and stability of the signals from the satellites to the output signal from the local oscillator, the local oscillator is controlled with a servo loop that compares and adjusts the phase of the reference signal from the GPS satellites to the phase of the signal from a voltage controlled ocsillator [13]. This way the local oscillator is permanently tied to an atomic clock and the GPSDO thus constitutes a “self-calibrating” frequency standard and a cost-effective alternative to an atomic clock. The frequency comb at the TU Ilmenau utilizes an oven-controlled crystal oscillator (OCXO) with a voltage-controlled frequency output of 10 MHz as GPSDO [57,58]. This output is fed to a signal distribution unit (SDU, manufacturer: Meinberg, 12 TTL output ports with 10 MHz, [59]) that disseminates the reference frequency to the control electronics and data acquisition system of the comb. A schematic of the OFC, the respective control electronics and data acquisition system is depicted in Figure 3.

The frequency comb itself is based on a mode-locked, polarization-maintaining erbium fiber laser with a center wavelength of 1552 nm and a default repetition rate of *f*_Rep_ = 250 MHz that can be adjusted between ± 1 MHz [60]. A 2*f*-*f* interferometer configuration is used for the detection of the CEO that is set to *f*_CEO_ = 20 MHz by default [60,61]. For the 2*f*-*f* interferometer, one part of the laser output is amplified in an erbium-doped fiber amplifier (EDFA) and afterwards spectrally broadened in a highly nonlinear fiber (HNLF). The frequency comb provides six output ports at 1550 nm with a maximum power output of 18 mW and a spectral width (FWHM) of 20 nm. Additionally, the comb is equipped with a second amplifier unit that generates a 633 nm output by spectral broadening in a HNLF and subsequent frequency doubling. This output provides an average power of 7 mW and a spectral width of 3 nm (FWHM). It can be used to create a beat signal with laser sources at 633 nm, e.g., He-Ne lasers. The data acquisition system of the comb is equipped with four Π-type frequency counters having a gate time of τ = 1 s (model: FXM50) [62] and two spectrum analyzers (model: HMS-X, Rohde & Schwarz). The frequency counters are referenced by the 10 MHz output of the SDU. The comb parameters *f*_Rep_ and *f*_CEO_ are monitored with two frequency counters. The remaining frequency counters can be used for the counting of beat frequencies with external laser sources. The feedback loops of the comb phase lock the repetition rate and the CEO to a multiple of the 10 MHz-frequency output [60]. A detailed description of the control loops can be found in [35,60]. An undisturbed reception of the GPS signal together with a permanent closed loop operation of the local oscillator are the basic prerequisite for a sufficient stabilization of the comb parameters and thus the generation of stable, traceable optical frequencies [14,35]. To control the locking status of the comb parameters, *f*_Rep_ and *f*_CEO_ are permanently measured by the frequency counters of the data acquisition system after the respective servo loops have been closed. For a continuous monitoring of the incoming GPS signals we extract data about the number of available satellites and the overall status of the GPS signals from the transmitted data files via the serial interface of the GPSDO. During a typical 24-h operation, we found that the number of available satellites usually varies between 6 and 10 which is sufficient for a distortion-free operation of the GPSDO. When proper operation of the GPDSO is assured, the manufacturer guarantees a relative accuracy better than 8 × 10^−12^ (τ = 1 s) and a relative frequency stability (relative Allan deviation) A_Dev_ better than 4 × 10^−12^ [57]. To determine the absolute frequency of external laser sources, the specific laser under test is superimposed with a comb line and the time dependence of the generated heterodyne signal is analyzed (see Section 2.4). For this purpose, the OFC is equipped with a free-space beat detection unit (FS-BDU) consisting of polarizing beam splitters, half-wave-plates, a diffraction grating and an avalanche photodiode (Model: APD210, Menlo Systems) for spatially overlapping the beams from the frequency comb and the external laser source (see e.g., [35]). For the detection of the heterodyne time traces, one of the counters of the data acquisition system is used.

#### 2.2.2. Optical Reference System (ORS)

**Figure 4:**
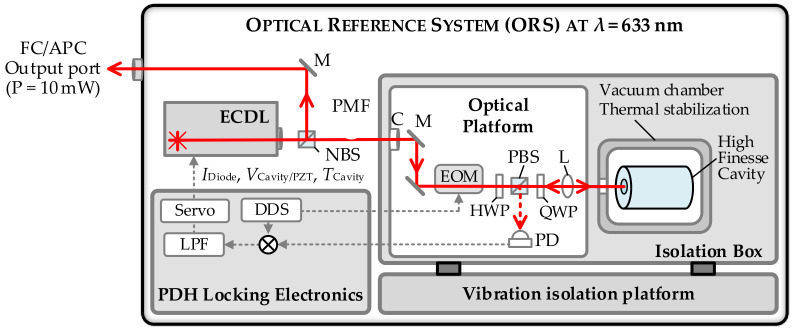
Structure of the ORS. The abbreviations denote: ECDL—external cavity diode laser, FC—fiber-coupled, NBS—neutral beam splitter, C—collimator, PMF—polarization-maintaining fiber, M—mirror, EOM—electro-optic modulator, HWP—half wave plate, PBS—polarizing beam splitter, QWP—quarter wave plate, L—lens, DDS—direct digital synthesizer, LPF—low pass filter. The red lines indicate optical path lengths, the dashed gray lines electronic signals. The light of the ECDL is sent through a phase-modulator operating at 20 MHz to generate sidebands for the PDH-lock. The 20-MHz reference signal is provided by a DDS. The error signal is created by mixing the signal from the photodetector containing the reflected light from the cavity with the signal from the DDS and subsequent low-pass filtering. The frequency of the laser is stabilized via fast feedback to the current and slow feedback to the piezo-electric actuator controlling the external cavity length of the diode laser [65].

The optical reference system is an ultrastable laser system based on a CW laser stabilized to a high-finesse cavity. The cavity serves as a reference with an exceptional short-term frequency stability [63]. In contrast, their long-term stability is limited by frequency drifts due to aging and temperature fluctuations of the cavity [63,64]. At the IPMS, a commercial system from Menlo Systems is under operation since 2021 [65]. The basic setup of the ORS is depicted in Figure 4. The ORS consists of an external cavity diode laser (ECDL, manufacturer: MOGLabs, Littrow configuration; wavelength: 632.8 nm [66]) that is locked to a cylindrical cavity made of ultra-low expansion (ULE) glass ceramics via the Pound–Drever–Hall (PDH) locking scheme [67,68]. The ULE spacer has a length of 121 mm and is equipped with high-finesse ion beam sputtered ULE mirrors [69]. The ULE glass exhibits a linear shrinking drift rate of approximately 0.15 Hz/s [69]. The optical cavity is temperature stabilized and embedded in an ultra-high vacuum (UHV) system with an achievable pressure of 7 × 10^−9^ mbar. The optics required to couple the light from the ECDL into the cavity and the opto-electronical components needed for the implementation of the PDH-Lock (electro-optic modulator for generation of sidebands at 20 MHz, quarter-waveplate, half-wave plate, polarizing beam splitter, photodetector, see Figure 4) are mounted on a breadboard vertically attached to the vacuum chamber. The cavity and all opto-mechanical parts are placed in an acoustic isolation box, mounted on an additional vibration isolation platform [69]. A control unit contains all electronics necessary for operating, tuning and locking the laser as well as the electronics for the temperature stabilization of the cavity [69]. The ECDL, ULE-cavity with optical platform and high vacuum pump and the control electronics are integrated into a compact 19” rack. The system provides an output power of 10 mW that can be accessed via a fiber-coupled, polarization-maintaining output port [69]. The ORS can be used either as stand-alone system or as an optical reference for the OFC (see e.g., [70]).

**Figure 5:**
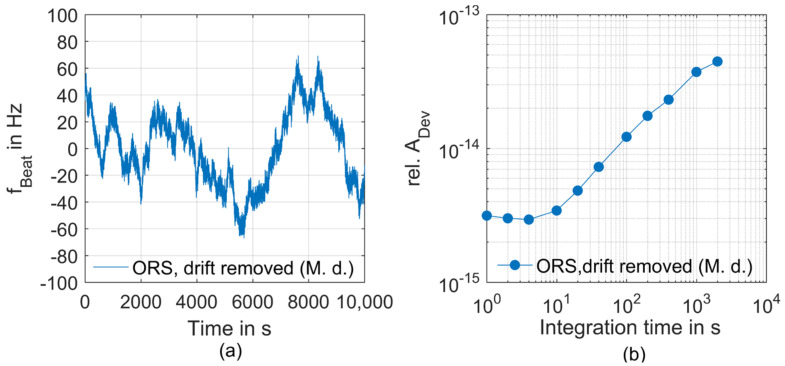
Frequency stability of the ORS as retrieved from manufacturer data (M.d.). (**a**) Time dependent beat frequency between two optical reference systems of comparable frequency stability with linear drift removed, (**b**) corresponding relative Allan deviation (see Section 2.4).

In Figure 5, the performance of the ORS-system as provided by the manufacturer is presented. In Figure 5a, the frequency deviation of the heterodyne beat signal between two optical reference systems of comparable frequency stability is presented. The data are already corrected for the drift rate of the cavity [71]. As can be seen from the corresponding relative Allan deviations depicted in Figure 5b, the ORS provides an excellent short-term stability of A_Dev_ = 3 × 10^−15^ at *τ* = 1 s [71]. Within the frame of the present work, we will apply the ORS as an ultrastable reference laser to characterize the short-term frequency instability of the OFC. This allows for the first time to directly resolve the instability behavior of our GPS-referenced frequency comb. For this purpose, the output of the ORS is heterodyned with one of the comb modes in a fully fiber-coupled beat detection unit (FF-BDU) [69].

### 2.3. Comb-Referenced, Fiber-Coupled He-Ne Laser Source

**Figure 6:**
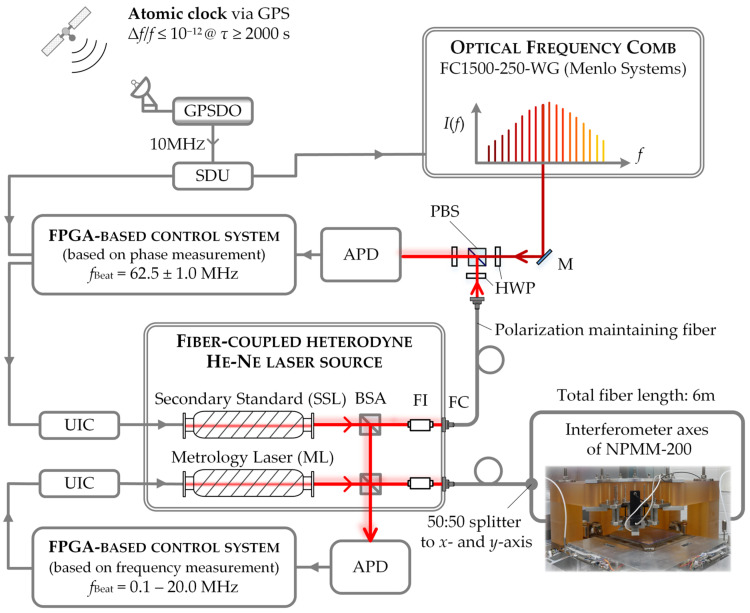
Layout of the comb-referenced He-Ne heterodyne source. The abbreviations denote: GPSDO—GPS disciplined oscillator, SDU—signal distribution unit, APD—avalanche photodiode, PBS—polarizing beam splitter, M—mirror, BSA—beam splitter assembly, FI—Faraday isolator, FC—fiber coupling, UIC—transconductive amplifier (U/I converter), FPGA—field programmable gate array.

For an easy integration of a comb-referenced metrology laser into the current length measurement system of the NPMM-200, we decided to build-up a He-Ne source similar to the laser module presented in Figure 1. Nevertheless, due to the limited bandwidth of the Ne gain profile, the approach of locking three independent lasers to individual comb lines as applied in comb-referenced multiwavelength interferometry is not a suitable approach for the He-Ne lasers used within the NPMM-200. Instead, we decided to implement a laser-array configuration as originally described in [72]. The laser module of the NPMM-200 was thus extended with a fiber-coupled digitally frequency offset-locked He-Ne heterodyne source consisting of two commercially available He-Ne lasers (model: SL02/1, SIOS Meßtechnik GmbH [73]) with the same mechanical design and similar transfer behavior [74]. The basic control concept of this heterodyne source is indicated in Figure 6 and was described in detail in [74]. The heterodyne source consists of two sealed internal mirror He-Ne laser tubes that are covered by a protective aluminum chassis and can be independently stabilized by a two-mode comparison technique using [74]. The respective optics and control electronics are integrated into the aluminum chassis and are provided by the manufacturer [73]. Both lasers are mounted on a U-profile which holds the beam splitters for beat frequency generation, Faraday isolators to to prevent back reflections into the laser tubes, fiber couplers, and the polarization maintaining optical fibers. Prior to fiber coupling, the lasers provide an output power of approximately 1.2 mW [73]. To adjust the frequency offset between the two lasers, some µW optical power are branched off with beam splitters to generate a beat signal with high SNR of approximately 50 dB. The beat signal is detected with an avalanche photo diode (Hamamatsu C5658). Due to the high SNR of the beat signal, a conventional edge-detection method can be used for frequency determination [74,75]. For this purpose, the analog signal from the photodiode is transformed into a TTL-signal via a Schmitt–Trigger circuit and is subsequently fed into an FPGA-based control loop (card: PXI-7853R and chassis PXI-1042Q). Therein, the number of counted pulses within a well-defined time frame is compared to a user defined frequency determining the frequency offset between the two lasers. The resulting frequency difference is fed into a digital PID-controller and the digital output signal is converted into an analog voltage [74,75]. The analog voltage is finally transformed into a current by a transconductance amplifier (U/I converter (UIC)) and drives the heater of the second laser tube to adapt its cavity length to follow the frequency of the first laser [74,75]. If the first laser is kept in its two-mode frequency stabilization regime, the maximum frequency deviation over one day limits the frequency stability of the heterodyne source to 2 × 10^−9^ (see Section 3.2).

To realize a comb-referenced He-Ne source, the first laser of the heterodyne-source has to be permanently locked to a single comb line. This is schematically shown in Figure 6. Since the optical power per comb line is low (approx. 800 nW per comb mode before passing optical components for beat signal detection), almost the entire output power of the first laser is used for generating a beat note with a respective comb mode [35]. The resulting beat signal with an SNR of 32–34 dB prevents the application of an edge-detection method. Thus, a phase-sensitive detection is applied for the frequency determination of the beat signal. For this purpose, the beat signal detected with an avalanche photodiode is fed to the analog input port of an FPGA-based control system (controller: NI-7931R and transceiver module: NI-5782). The input signal is converted by an analog-to-digital converter (ADC) of the control system with a sampling rate of up to 250 MS/s and is further processed by the embedded FPGA unit (model: Xilinx Kintex-7-K325T). The control electronics are synchronized via the 10-MHz output of the GPSDO. The implemented servo loop allows to correctly determine the beat frequency even at a low SNR ≤ 10 dB. The detection algorithm is currently designed with a ± 1-MHz bandpass filter around a fixed center frequency of 62.5 MHz [75]. The phase data of the input signal is used to generate a frequency variable by subtracting subsequent values over a time slot of 64 ns. The resulting frequency value is low-pass-filtered and finally forwarded to a PID controller algorithm [75]. The resulting digital output signal is afterwards converted into a DC coupled voltage signal using a digital-to-analog-converter (DAC) with a resolving capability of 20 bit (model: AD5791, Analog Devices) [75]. Finally, the voltage signal is converted into a current by a second transconductive amplifier. This current drives the heating coil of the first laser of the heterodyne source to change its resonator length and thus adapting its frequency to follow the frequency of the respective comb line [75].

This way, the He-Ne laser directly stabilized to a comb line serves as a “Secondary Standard Laser” (SSL) as depicted in Figure 6 and the second laser of the heterodyne-source tied to the SSL serves as the actual metrology laser (ML). The specific locking procedure and determination of the absolute frequency of both laser sources is described in detail in [35]. The remaining optical power of 500–700 µW from the metrology laser is finally used to feed the two 200-mm interferometer axes (x and y) of the NPMM-200. In opposition to the original configuration shown in Figure 1, this allows to transfer the same laser frequency to the *x*- and *y*-interferometer axes of the NPMM-200. To disseminate the comb-stabilized frequency output to the interferometers of the NPMM-200 a polarization maintaining (PM) 50:50 fiber coupler and a 6 m polarization-maintaining fiber is used.

### 2.4. Analysis of Frequency Instability

For the analysis of the frequency instability of the GPS-referenced frequency comb as well as the OFC-locked He-Ne lasers, we use the heterodyne method. For this purpose, the GPS-referenced frequency comb is superimposed with the ORS or the He-Ne lasers within the FF-BDU or the FS-BDU as described within the prior sections. Subsequently, the respective beat signals of the APD are low-pass filtered, amplified, and band pass filtered (3-dB bandwidth 49.8 MHz… 70.5 MHz around the center frequency of 60 MHz, [76]). The bandpass-filtered signal is fed to the frequency counters and the recorded frequency-over-time-traces are used to analyze frequency instability in the time domain. To study frequency instability in time-domain, the Allan variance $\sigma_{y}^{2}\left(\tau \right)$ is calculated for a discrete, finite series of *N* frequency values *f_i_* averaged over a specific sampling time *τ* that can be obtained as an integer multiple *m* of a basic measurement interval *τ*_0_ [77,78]:

(2) $$\sigma_{y}^{2}\left(\tau \right)=\frac{1}{2\left(N-1\right)}\sum_{i=1}^{N-1}{\left[f_{i+1}-f_{i}\right]}^{2}.$$

The basic measurement interval is determined by the gate time of the FXM50 frequency counter used throughout this work and corresponds to *τ*_0_ = 1 s. For practical calculations of the Allan variance, usually overlapping samples are used to improve the confidence of the stability estimate and the results are expressed as the square root of $\sigma_{y}^{2}\left(\tau \right),$ the Allan deviation A_Dev_ [78,79].

## 3. Results and Discussion

### 3.1. Frequency Instability of the GPS-Referenced Frequency Comb

**Figure 7:**
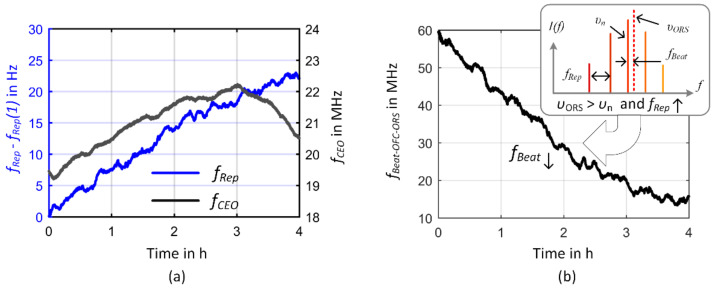
Frequency instability of the free-running comb. (**a**) Time traces of the free-running comb parameters measured with the FXM50. The left *y*-axis depicts the change of repetition rate from its initial value. The right *y*-axis the CEO. (**b**) Time trace of the beat note between the respective comb line and the ORS. Since the frequency υ_ORS_ of the ORS lies above the respective frequency of the comb line υ_n_, an increase in the repetition rate *f_Rep_* results in a decrease of the beat frequency *f_Beat_*. To monitor the frequency changes of the beat note, the measurements were performed without the bandpass filter at 60 MHz.

**Figure 8:**
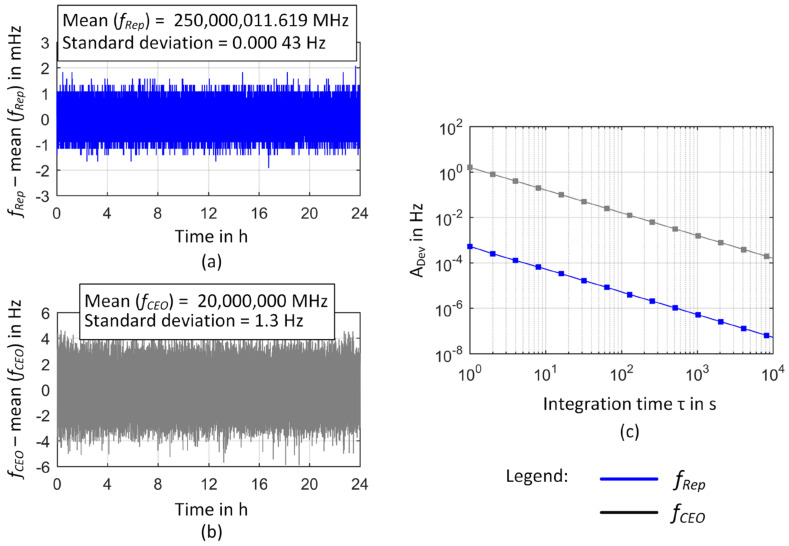
In-loop comb parameters of the GPS-referenced frequency comb. (**a**) Time trace of the locked repetition rate. (**b**) Time trace of the locked CEO. Both plots depict the frequency deviations from the mean value over a measurement time of 24 h. (**c**) Calculated Allan deviations. The gray and blue lines present a linear fit to the data as described within the text.

**Figure 9:**
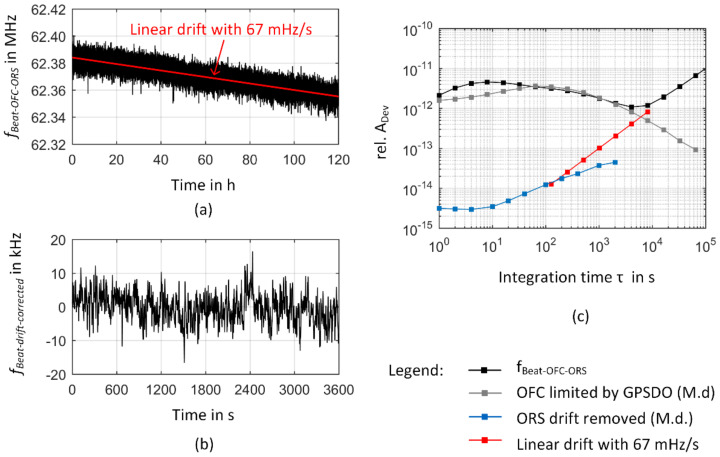
Frequency instability of an in-loop GPS-referenced frequency comb. (**a**) Time trace of the heterodyne signal between the OFC and the ORS. The red line indicates a linear fit to the data with *f* = 62.384 MHz–0.067 Hz/s. (**b**) A 1 h section of the frequency fluctuations of the heterodyne signal after the linear drift was removed from the measured data. (**c**) The respective relative Allan deviation of the heterodyne signal obtained by dividing the Allan deviation by *f*_HeNe_ ≈ *f*_Comb_ ≈ 474 THz. The abbreviation M.d. denotes “manufacturer data”.

The time traces of the frequency measurements of the comb parameters and the heterodyne signal between the respective comb line and the ORS are shown in Figure 7 for the free-running comb over a 4-h measurement interval and in Figure 8 and Figure 9 for an in-loop operation of the comb. For better readability, the changes in repetition rate in Figure 7a are depicted as deviation from its initial value. The free-running repetition frequency shows a slight drift of 22 Hz within the observed time window. Although the respective changes in the CEO within the same observation time are within the MHz-range, the resulting frequency change of the respective comb line is completely determined by the change in repetition rate as can be deduced from the frequency drift of the beat frequency depicted in Figure 7b. Due to the large multiplication of the repetition frequency into the optical domain as described by Equation (1), the rather small frequency drift of the repetition rate results in a 42 MHz (Δ*f*/*f* = 8.9 × 10^−8^ with *f*_HeNe_ = 473.6127 THz) frequency change of the respective comb mode (*n* = 1,894,861) that is reflected in the frequency drift of the beat frequency between the comb line and the ORS. The opposite sign of the slope is caused by the sign of the beat signal. Since the ORS frequency lies above the considered comb line, an increase in the repetition rate leads to a decrease of the beat frequency as demonstrated in the inset of Figure 7b.

In Figure 8a,b, the time traces of *f*_Rep_ and *f*_CEO_ locked to the rf reference are presented. Both comb parameters follow the reference frequency provided by the GPSDO with a maximum frequency deviation from the mean value of 2 mHz (Δ*f*/*f*_Rep_ = 8 × 10^−12^) for *f*_Rep_ and 6 Hz (Δ*f*/*f*_CEO_ = 3 × 10^−7^) for *f*_CEO_. The respective Allan deviations in Figure 8c present the tracking stability of the comb parameters with a *τ*^−1^-dependence for both parameters. This corresponds to white phase noise characteristics as supported by the modified Allan deviation showing a *τ*-^−3/2^-dependence (see e.g., [78]) for integration times below 10,000 s (not shown Figure 8c).

To analyze the resulting frequency instability of the respective comb line, the time trace of the heterodyne signal between the OFC and the ORS and the respective relative Allan deviation are presented in Figure 9. In Figure 9a, a clear frequency drift is evident when measuring the beat frequency over a period of 5 days. This frequency drift is mainly caused by the frequency drift of the cavity material of the ORS. To a first approximation it can be described by a linear fit with a slope of 67 mHz/s, resulting in a frequency change of 5.8 kHz (Δ*f*/*f* = 1.2 × 10^−11^, *f* = 474 THz) in 24 h and an overall frequency drift of 28.9 kHz within the observation time of 5 days. The frequency drift of the ORS cavity thus dominates the long-term stability of the heterodyne signal as can be deduced from the increase of the Allan deviation for *τ* > 4000 s in Figure 9c and the data points of the Allan deviation of a linear drift rate introduced as a red line in Figure 9c. Nevertheless, the observed drift rate is much smaller than the drift rate of 150 mHz/s as reported by the manufacturer [69]. This can be attributed to aging effects of the cavity material as reported in [64] and is consistent with prior measurements where a drift rate of 104 mHz/s was obtained [80].

Comparing the Allan deviation of the beat signal with the data for the ORS as given by the manufacturer thus clearly reveals that the Allan deviation for *τ* < 4000 s reflects the frequency instability of the GPS-referenced frequency comb. Therefore, the heterodyne signal mainly represents frequency fluctuations of the respective comb line when the drift rate is removed as indicated in Figure 9b. Within the measurement interval of 5 days, a maximum frequency deviation of 25.2 kHz (Δ*f*/*f* = 5.3 × 10^−11^) was observed from the drift-corrected frequency values of the beat signal. Over a timeframe of 1 h, a typical maximum frequency deviation of 14.8 kHz (Δ*f*/*f* = 3.1 × 10^−11^, obtained as a mean value of 1-h sections within the 5-day time frame for *τ* = 1 s) occurred. The measurement data of the unlocked comb presented in Figure 7 demonstrated the influence of small frequency changes of the repetition rate on the overall frequency instability of a comb line. Since the measurement of the comb parameters revealed a high tracking stability (Figure 8), the Allan deviation of the heterodyne signal between the comb line and the ORS thus essentially reflects the frequency instability of the GPSDO. For comparison, Figure 9 additionally contains Allan deviation data provided by the manufacturer (gray line in Figure 9c). This dataset was obtained from a 13-h measurement series of a comb–comb comparison with two GPS-referenced frequency combs [57]. For short integration times, the Allan deviation increases with a slope of *τ*^0.2^, reaching a distinct maximum of 3.6 × 10^−12^ at *τ* = 64 s. For longer integration times, the Allan deviation decreases. This behavior is consistent with former results reported in the literature [81,82,83,84]. The frequency output of the OCXO is steered to agree with the signals transmitted by the GPS satellites and can thus take advantage of their high long-term stability [13,84].

The Allan deviation obtained from the frequency measurement between a comb line and the ORS starts to coincide with the manufacturer data at *τ* = 64 s. For smaller integration times, considerably higher Allan deviations are obtained from the ORS-OFC comparison. In opposite to the manufacturer data a maximum Allan deviation of 4.6 × 10^−12^ at *τ* = 8 s is observed. The origin of this discrepancy is currently not clear. We did not find any indications that the settings of the comb parameter control loops (e.g., a too high P-gain) or the additional integration of the SDU led to these differences. Therefore, further investigations are needed to clarify the origin of this degradation in short-term-frequency stability compared to the manufacturer data. Since the accuracy of the GPSDO over a given period of time is usually limited by its stability, it is common sense to use twice the Allan deviation for a time duration that orientates towards the duration of frequency calibration to determine the frequency uncertainty of a GPSDO [82]. Covering a worst-case scenario, manufacturer data rather tend to “overestimate” uncertainty by relying on the highest Allan deviation reported (see Section 2.2). Taking these considerations into account and regarding that the frequency instability of a comb line is primarily determined by the frequency instability of the used rf reference (see e.g., [52]), we have to claim an uncertainty of 9.2 × 10^−12^ (k = 2, *τ* = 8 s) for the GPS-referenced frequency comb at the TU Ilmenau. Therefore, reaching an uncertainty below 10^−12^ makes an integration time of at least 8000 s necessary.

### 3.2. Frequency Instability of the Comb-Referenced He-Ne Source

**Figure 10:**
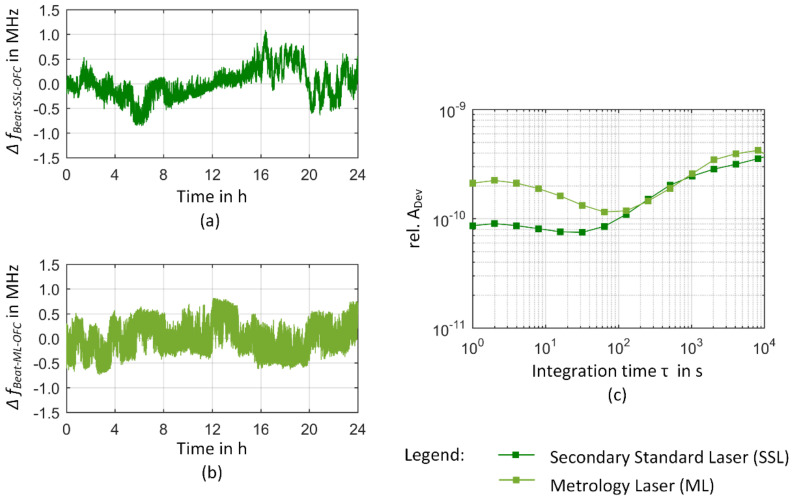
Frequency instability of the He-Ne heterodyne source used as a starting point for the realization of a comb-referenced laser module. (**a**) A 24 h-measurement series of the secondary standard laser (SSL) against a comb line, (**b**) 24 h measurement of the metrology laser (ML). In each of the plots, the frequency deviations from the mean value derived over the whole measurement series of 24 h are shown. (**c**) Relative Allan deviations obtained by dividing the frequency fluctuations by a nominal value of 474 THz.

**Figure 11:**
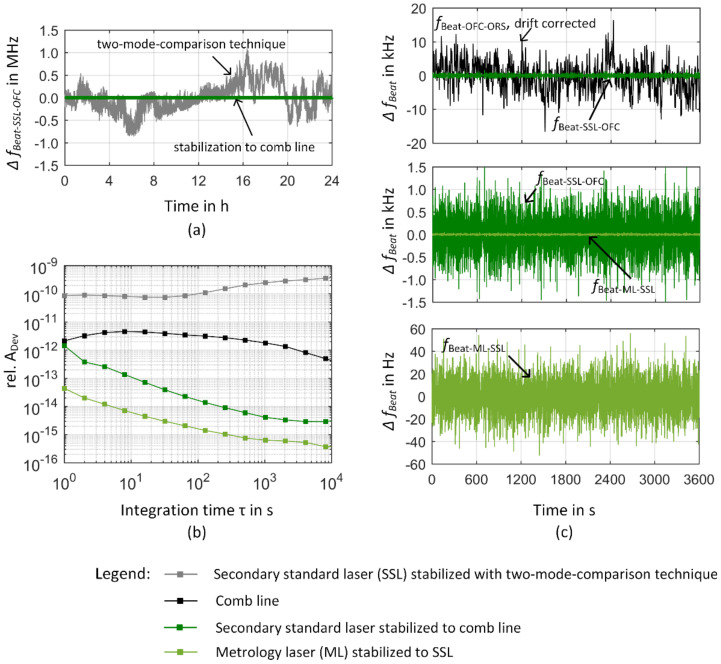
Frequency instability of the comb-referenced-He-Ne heterodyne source. (**a**) 24-h measurement series of the secondary standard laser (SSL) prior and after closing the control loop (closed-loop operation reproduced from [35]). (**b**) Relative Allan deviations. The Allan deviation of the OFC is obtained by combining the Allan deviation as calculated from the measurement of the beat signal between the ORS and the OFC for integration times below 4000 s and the Allan deviation given by the manufacturer for integration times above 4000 s. (**c**) The 1 h measurement sections of the 24-h timetrace. The upper graph depicts the SSL locked to the comb and the frequency fluctuations of the drift-removed beat signal between a comb line and the ORS (taken from Figure 9b) for comparison. Please note that in contrast to the in-loop measurements of the heterodyne source, those measurements were not taken simultaneously. The graph in the middle enlarges the frequency scale to illustrate the frequency fluctuations of the SSL when locked to the comb line. The lower graph further enlarges the frequency scale to depict the fluctuations of the beat signal between the ML and the SSL, when locked to the comb line. All three graphs show the same 1 h section and present the frequency deviations from the respective mean value of the whole measurement series.

In Figure 10 and Figure 11, the frequency stability of the comb-referenced He-Ne sources is finally presented. Figure 10 presents a measurement series of the He-Ne heterodyne source when both lasers are in their internal two-mode comparison stabilization regime and are measured against an optical comb line. In this case, the comb serves as a stable reference laser revealing the frequency instability characteristics of the He-Ne heterodyne source. In Figure 10, the frequency fluctuations over a typical measurement interval of 24 h obey a long-periodic frequency change that is superimposed by several frequency jumps up to 1.5 MHz. The long-term frequency changes lead to a rise of the Allan deviation for *τ* > 100 s resulting in a deviation of 3–4 × 10^−10^ at *τ* = 8000 s. The first laser serving as a secondary standard laser (SSL) obeys a maximum frequency deviation of 1 MHz. Although both lasers are identical in construction and their long-term frequency instability is comparable as deduced from the intersection of the Allan deviation for *τ* > 100 s, they obey a different frequency stability behavior within the short-term regime as can be seen from the frequency fluctuations in Figure 10a,b as well as the Allan deviation below 100 s. Nevertheless, the frequency instability of both lasers is consistent with the results we recently reported for the lasers of the He-Ne module of the NPMM-200 [35]. Thus, the heterodyne source can be considered as a suitable representative of the frequency characteristics of the NPMM-200 laser module and an appropriate candidate for the realization of a comb-referenced laser module.

The final closed loop operation of the comb-referenced He-Ne source is depicted in Figure 11. In Figure 11a, the deviation of the beat frequency between the SSL and a comb line from its mean value Δ*f*_Beat_ = *f*_Beat_ − $\bar{f_{Beat}}$ after closing the control loop is shown. Additionally, the changes of the heterodyne signal when the SSL is in its internal stabilization regime are plotted for comparison from Figure 10a. When the control loop between the comb line and the SSL is closed, the frequency deviations of the SSL are eliminated and the beat signal reflects the tracking stability of the SSL in relation to the comb line [75]. In Figure 11c, a 1 h-section of the measurement series is enlarged. The upper graph of Figure 11c additionally contains the changes of the beat signal of the comb line obtained from the measurement data of the beat frequency between a comb line and the ORS as discussed in Section 3.1. Please note that these two measurements were not taken simultaneously and their absolute frequencies correspond to two different comb lines due to the different absolute frequencies of the ORS and the He-Ne lasers.

In the central and lower graph of Figure 11c, this 1-h section is further enlarged to make the frequency deviations of the beat signals between the SSL and the comb line as well as the ML and the SSL visible when both control loops were closed. Therein, the bright green line represents the frequency deviation from the mean value of the beat signal between the SSL and the ML and thus demonstrates how the ML is following the SSL when the SSL is locked to the comb line. Considering the whole 24-h measurement series occasional outliers resulting in a maximum frequency deviation of 11.7 kHz were observed [35]. These outliers stem from severe acoustic noise due to slamming of doors and are to be avoided within a normal operation of the comb-referenced He-Ne lasers and the NPMM-200. The 1-h section in Figure 11c presents a part of a 10-h measurement window where no extreme external disturbances occurred. Based on this dataset, we observed on average a maximum frequency deviation of 1.8 kHz (Δ*f/f* = 3.8 × 10^−12^ with *f* = 474 THz) with a standard deviation of 146 Hz for the in-loop heterodyne signal between the SSL and the comb line and of 62 Hz (Δ*f*/*f* = 1.3 × 10^−13^) with a standard deviation of 5 Hz for the in-loop heterodyne signal between the SSL and the ML within a 1 h measurement window (values are obtained as a mean value of 1-h sections within the 10-h measurement window for *τ* = 1 s). On the other hand, the deviations of the mean value of the measured beat frequencies from the nominal values of the closed-loop control are −47 Hz (Δ*f*/*f* = 9.9 × 10^−14^) at *f*_0_ = 62.5 MHz for the SSL and 204 Hz (Δ*f*/*f* = 4.3 × 10^−13^) for the ML at *f*_0_ = 4.5 MHz [35]. Comparing this to the observed maximum frequency deviations from the mean value, it can be deduced that the accuracy of the ML control loop is limited by a frequency offset and not the frequency stability of the beat signal.

**Figure 12:**
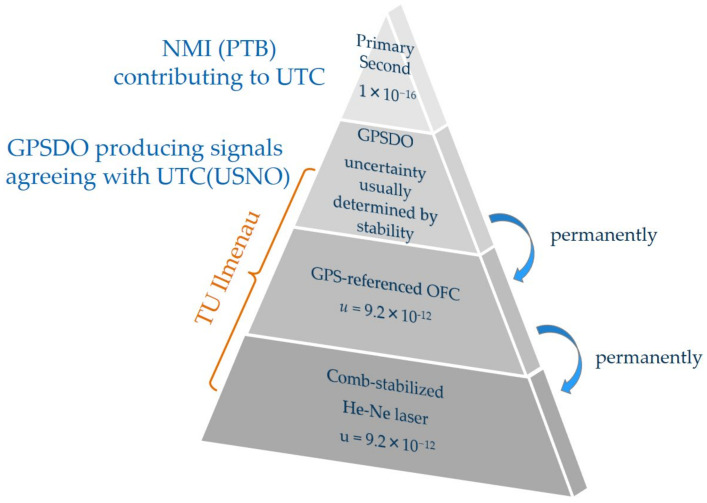
Scheme of the reduced traceability chain for laser wavelength calibration of the metrology lasers of the NPMM-200.

The corresponding relative Allan deviations of the measurement series are presented in Figure 11b. For a better comparison of the frequency instability of the in-loop beat signals to the Allan deviation of the comb line, all values were related to a nominal frequency of 474 THz. The black curve depicts a combination of the Allan deviation of the frequency comb as obtained from the measurement of the beat signal between the ORS and the OFC for integration times below 4000 s and the manufacturer data for integration times above 4000 s. The relative Allan deviation of the beat signals between the SSL-OFC and the ML-SSL both fall below the Allan deviation of the comb line for integration times *τ* ≥ 1 s. This again demonstrates that both servo loops enable the He-Ne lasers of the heterodyne source to follow the comb line. The Allan deviation of the beat frequency between the SSL/comb line (SSL/ML) fall with a slope of *τ*^−0.8^ (*τ*^−0.6^) for integration times between 2 s and 500 s that flattens down to a *τ*^−0.2^–dependence for integration times above 1000 s. The frequency stability of the comb-referenced metrology laser is thus finally determined by the Allan deviation of the comb line. The frequency fluctuations and differences in the Allan deviation of the beat signals between the SSL/comb line and the SSL/ML furthermore indicate that the limited and similar dynamic behavior of the laser control systems produces some lowpass filtering effects on the higher frequency distortions of the comb line. In this case, the locked SSL would not be able to follow fast comb line jitters leading to a “smoothing” out of frequency distortions of the comb line [75]. To prove this thesis, we now prepare direct measurements of the He-Ne-locked system against the ORS and concentrate on more detailed investigations on the frequency noise properties of the comb line and the in-loop-comb-referenced metrology laser. Finally, comparing the Allan deviation of the comb line with the Allan deviation of the SSL that represents the previous status of frequency stability in the NPMM-200, an improvement of nearly two orders of magnitude at *τ* = 1 s and of up to three orders of magnitude at *τ* = 10,000 s can be achieved. Thus, the approach of a comb-referenced He-Ne laser source allows a significant improvement of the long-term frequency stability of these lasers and additional changes in absolute frequency due to aging effects can permanently be monitored and avoided once their absolute frequency has been determined as described in [35]. As a result, the traceability chain for the measurement of laser frequency is significantly changed in comparison to Figure 2. The NMIs contribute to the calculation of the UTC by sending data from their local clocks to the BIPM [82]. The GPS satellites carry atomic oscillators and are controlled by the United States Department of Defense (U.S. DoD) [13]. The GPS time is referenced to the UTC time scale maintained at the United States Naval Observatory UTC (*USNO*) which is the largest contributor to the calculation of the UTC [82]. This way, the GPS receivers generate output signals that agree with UTC (*USNO*) [82]. Within the GPSDO, the output signals from the GPS receiver are used to discipline the local oscillator providing a frequency output with an uncertainty that is mainly determined by the frequency instability of the locked oscillator [13,82]. The comb technology allows to transfer the accuracy and stability of the GPSDO into the optical domain, where it is finally delivered to the length measuring system of the NPMM-200 by the comb-referenced He-Ne source. Thus, a direct and permanent link to the SI unit second can locally be established as schematically shown in Figure 12.

Nevertheless, it has to be mentioned that the current locking scheme does not compensate any potential frequency fluctuations due to environmental disturbances within the fiber link transporting the frequency of the comb-referenced metrology laser to the length measurement system of the NPMM-200 as discussed in Section 2.3. Since these disturbances are mainly caused by thermal noise and their impact increases with fiber length, we chose the fiber length as short as possible (see e.g., [85] for fiber thermal noise). The comb and the heterodyne source are placed in the same air-conditioned lab as the NPMM-200 keeping the impact of temperature variations as small as possible. Based on a worst-case-scenario calculation retrieved from [86], where a maximum temperature change of 7 mK/s obtained from temperature data within the lab was assumed, a maximum frequency change of 8 × 10^−16^ per meter fiber length was retrieved [35]. Compared to the Allan deviation of our GPS-referenced frequency comb, this frequency change is neglectable for the 6-m fiber length for integration times below 10,000 s. Nevertheless, if prospectively longer fiber paths covering several ten meters of fiber length for a further internal distribution of the ultrastable frequencies are considered, the validity of our assumption has to be confirmed within further experiments on the phase-noise characteristics of the comb-referenced fiber-coupled He-Ne laser source. From the current point of view, the overall uncertainty of the comb-referenced metrology laser is thus determined by the frequency instability of the respective comb line for integration times up to 10,000 s.

**Figure 13:**
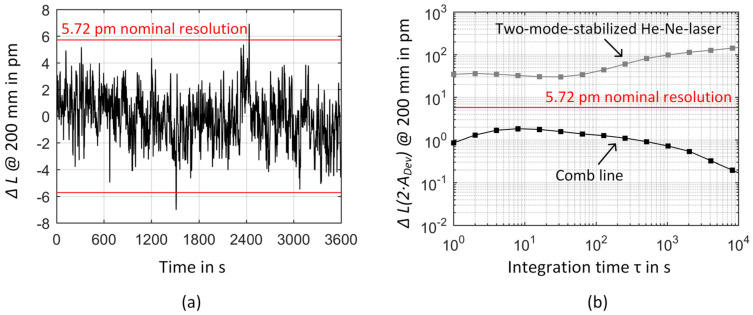

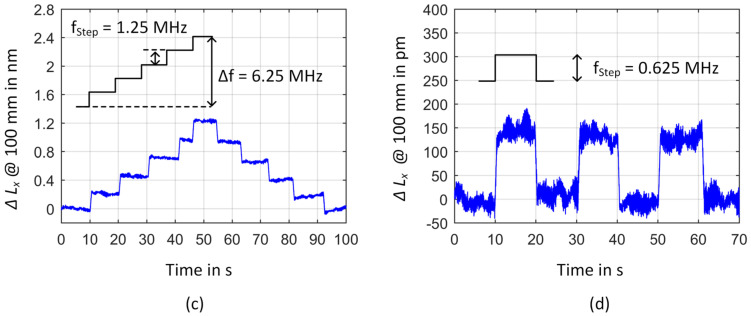
Influence of frequency distortions on the interferometric length measurement within the NPMM-200. (**a**,**b**) transform the frequency fluctuations and uncertainty of the comb line into a theoretical length measurement error for a maximum measurement range of *L* = 200 mm. (**c**,**d**) experimentally demonstrate the influence of frequency changes on the interferometric length measurement within the NPMM-200 at a constant distance of *L* = 100 mm using the comb-referenced He-Ne laser as a tunable laser source, (**c**) equidistant frequency steps of Δ*f* = 1.25 MHz and (**d**) repeated frequency jumps of Δ*f* = 0.625 MHz height. Integration time 0.5 s [35].

In Figure 13, finally the influence of frequency instability on the length measurement within the NPMM-200 is discussed. In Figure 13a,b, the expected frequency fluctuations of the comb line obtained from the heterodyne signal between the OFC and the ORS are transferred into a length measurement error using the interferometer equation for a Michelson interferometer configuration [87]:

(3) $$L=\frac{N\cdot c}{2\cdot e\cdot n\cdot f}$$

where *L* describes the measured displacement, *N* the respective counter value that corresponds to a change in interference order, *c* the velocity of light, *e* an electronic interpolation factor, *n* the refractive index of the ambient medium, and *f* the frequency of the laser source. By applying mathematical differentiation on Equation (3) with respect to frequency and subsequent dividing by *L* to obtain a relative expression on the resulting length measurement error one obtains:

(4) $$\frac{\Delta L}{L}=-\frac{\Delta f}{f}$$

In Figure 13, Equation (4) is used to calculate Δ*L* for a maximum measurement range of *L* = 200 mm where for simplicity the absolute value of Δ*f*/*f* is used. As can be seen in Figure 13a, even regarding a worst case scenario where a complete transfer of frequency fluctuations of the comb line to the He-Ne laser is assumed, the fluctuations of the comb line mainly stay below the nominal resolution of the current signal processing unit of the NPMM-200. In Figure 13b, the expected length measurement error was calculated based on the uncertainty of the comb as discussed in Section 3.1. Therein, a maximum length measurement error of only 2 pm is expected for *L* = 200 mm demonstrating that the influence of frequency distortions fall below the nominal resolution of the current interferometers within the NPMM-200 for integration times above 1 s and can thus be neglected. This is also true if the additional tracking instability of both beat signals would be added to the relative Allan deviation values underlying this calculation. For comparison, Figure 13b additionally plots the length measurement error obtained from twice the Allan deviation of the He-Ne laser when stabilized by the two-mode comparison technique. This presents the former status within the NPMM-200 and demonstrates the not negligible influence of the long-term stability on the length measurement. Nevertheless, the negligible length measurement errors expected from the comb-referenced He-Ne lasers makes a direct experimental study on their impact within the NPMM-200 difficult. Therefore, as an indirect proof of our approach, we used the tunability of the comb-referenced He-Ne source to directly demonstrate and separate the influence of frequency distortions on the interferometric length measurements. For this purpose, we intentionally introduced frequency changes by varying the beat frequency between the ML and SSL when both lasers are locked to the comb line. For this measurement, the positioning stage of the NPMM-200 was mechanically fixed at a distance of L = 100 mm and the drive system of the NPMM-200 was deactivated. The measurements were taken under normal atmospheric conditions without evacuating the NPMM-200 chamber. During the measurements either equidistant frequency steps or frequency jumps where applied. The absolute value of the frequency changes was adapted to correspond to the typical frequency changes we observed when the He-Ne lasers are in their internal stabilization mode based on the two-mode comparison technique as discussed in Figure 10. The results of these measurements are presented in Figure 13c,d. According to Equation (4), the subsequent frequency steps of 1.25 MHz in Figure 13c should lead to a frequency-related length change of 264 pm and in Figure 13d repeated, individual frequency jumps of 625 kHz should cause a length change of 132 pm for the considered measurement range of 100 mm. Experimentally, we observed a mean length change of 0.25 nm with a standard deviation of 0.03 nm per frequency step for the frequency staircase, resulting in an overall frequency change of 6.25 MHz that corresponds to a frequency related length change of 1.3 nm as clearly visible in Figure 13c [35]. The deviations from an ideal staircase observed in Figure 13c indicate that disturbances are not only caused by the introduced frequency changes but also contain mechanical vibrations, temperature fluctuations, and related changes in the refractive index making a baseline correction of the data necessary. In case of the up-and-down frequency jumps, an average length change of 133.5 pm with a standard deviation of 9.9 pm was retrieved from the measured data after averaging down the measured length data with an integration time of 0.5 s [35].

## 4. Summary and Outlook

Ultrastable and traceable wavelength standards are a key component for high-precision displacement interferometry in nanopositioning and nanomeasuring machines. With the demand for extended measurement volumina, the respective wavelength standards have to meet increasingly higher demands as well. Based on the state-of-the-art length measuring system of the NPMM-200 with a measurement volume of 200 mm × 200 mm × 25 mm, we deduced the requirements for such a wavelength standard and described how it was experimentally implemented by means of a comb-referenced He-Ne heterodyne source at 633 nm. The key component, a GPS-referenced frequency comb, serves as a “self-calibrating” frequency standard providing direct traceability to the SI unit second and an exceptional long-term frequency stability of 5 × 10^−13^ at *τ* = 10,000 s. By locking the He-Ne lasers to a comb line, it was possible to improve their long-term frequency stability by three orders of magnitude. This approach allows us to establish a traceable and permanent link of interferometric length measurements to the SI unit second. With the improved frequency stability of the comb-referenced gas lasers, the influence of frequency-related length measurements errors practically drops below the nominal resolution of the signal processing unit of the NPMM-200 for the first time. This allows to neglect the laser frequency as an uncertainty contribution factor within our length measurements.

However, our experiments also revealed limitations of the implemented GPS-referenced wavelength standard. The comparison of the GPS-referenced frequency comb against an optical reference system with an outstanding short-term frequency stability of Δ*f*/*f* = 3 × 10^−15^ at τ = 1 s enabled to directly resolve the short-term frequency instability of a single comb line that is limited by the frequency instability of the underlying GPSDO. Based on this comparison, a maximum relative Allan deviation of Δ*f*/*f* = 4.6 × 10^−12^ at *τ* = 8s was obtained demonstrating the typical limitations of a GPS-referenced frequency comb that makes a frequency stability better than Δ*f*/*f* = 1 × 10^−12^ only accessible for integration times longer than *τ* = 8000 s. Furthermore, the currently used He-Ne heterodyne source provides only a limited output power thus lowering the signal-to-noise ratio of the interferometer signals in comparison to the prior interferometer configuration with three independent He-Ne lasers. Our upcoming experiments will thus focus on a characterization of the frequency noise properties of the comb-referenced He-Ne heterodyne source with respect to comb line frequency noise and fiber dissemination to clarify if the high-frequency distortions of the comb line also enhance the frequency noise of the currently used He-Ne lasers. Future experiments will also have to enhance the currently limited output power of the GPS-referenced wavelength standard to feed all of the interferometer axes of the NPMM-200. Although the number of comb-referenced He-Ne lasers is easily scalable within the current control system, it is questionable if in terms of different noise properties of the He-Ne sources, the build-up of an extended laser array is desirable. Therefore, we will concentrate on directly integrating the ORS as an ultrastable, high-power laser source into the length measuring system of the NPMM-200. For this purpose, a suitable procedure for a correction of the cavity drift by means of the GPS-referenced frequency comb has to be established. In perspective, the combination of the ORS and the GPS-referenced OFC will allow to establish a wavelength standard of high output power providing a frequency stability of better than Δ*f*/*f* = 1 × 10^−12^ independent on integration time to be used for displacement interferometry within NPMMs up to 1 m or a further on-site dissemination of ultrastable optical frequencies for the implementation of comb-based interferometry approaches within NPMMs for, e.g., refractive index correction.

## Data Availability

Research data available upon request to the authors.
